# Continuing versus Stopping Prestroke Antihypertensive Therapy in Acute Intracerebral Hemorrhage: A Subgroup Analysis of the Efficacy of Nitric Oxide in Stroke Trial

**DOI:** 10.1016/j.jstrokecerebrovasdis.2016.01.010

**Published:** 2016-05

**Authors:** Kailash Krishnan, Polly Scutt, Lisa Woodhouse, Alessandro Adami, Jennifer L. Becker, Lesley A. Cala, Ana M. Casado, Christopher Chen, Robert A. Dineen, John Gommans, Panos Koumellis, Hanna Christensen, Ronan Collins, Anna Czlonkowska, Kennedy R. Lees, George Ntaios, Serefnur Ozturk, Stephen J. Phillips, Nikola Sprigg, Szabolcs Szatmari, Joanna M. Wardlaw, Philip M. Bath

**Affiliations:** * Stroke Trials Unit, Division of Clinical Neuroscience, University of Nottingham, Nottingham, United Kingdom; † Stroke Centre, Ospedale Sacro Cuore Via Sempreboni, Verona, Italy; ‡ Department of Medical Imaging, College of Medicine, The University of Arizona, Tucson, Arizona; § School of Pathology and Laboratory Medicine, The University of Western Australia, Nedlands, Australia; ‖ Division of Neuroimaging Sciences, Centre for Clinical Brain Sciences, Western General Hospital, Edinburgh, United Kingdom; ¶ Department of Pharmacology, National University Hospital of Singapore, Singapore; # Radiological Sciences Research Group, Division of Clinical Neuroscience, University of Nottingham, Nottingham, United Kingdom; ** Department of Medicine, Hawke's Bay Hospital, Hastings, New Zealand; †† Department of Neuroradiology, Nottingham University Hospitals, Queen's Medical Centre, Nottingham, United Kingdom; ‡‡ Department of Neurology, Bispebjerg Hospital, Copenhagen, Denmark; §§ Stroke Service, Adelaide and Meath Hospital, Dublin, Ireland; ‖‖ Department of Neurology, Institute of Psychiatry and Neurology, Warsaw, Poland; ¶¶ Institute of Cardiovascular and Medical Sciences, University of Glasgow, Glasgow, United Kingdom; ## Department of Medicine, University of Thessaly, Larissa, Greece; *** Department of Neurology, Selcuk University Medical Faculty, Konya, Turkey; ††† Division of Neurology, Queen Elizabeth II Health Sciences Centre, and Department of Medicine, Dalhousie University, Halifax, Nova Scotia, Canada; ‡‡‡ Department of Neurology, Clinical County Emergency Hospital, Targu Mures, Romania

**Keywords:** Antihypertensive therapy, blood pressure, glyceryl trinitrate, intracerebral hemorrhage, cerebrovascular disorders, randomized controlled trial

## Abstract

**Background and purpose:**

More than 50% of patients with acute intracerebral hemorrhage (ICH) are taking antihypertensive drugs before ictus. Although antihypertensive therapy should be given long term for secondary prevention, whether to continue or stop such treatment during the acute phase of ICH remains unclear, a question that was addressed in the Efficacy of Nitric Oxide in Stroke (ENOS) trial.

**Methods:**

ENOS was an international multicenter, prospective, randomized, blinded endpoint trial. Among 629 patients with ICH and systolic blood pressure between 140 and 220 mmHg, 246 patients who were taking antihypertensive drugs were assigned to continue (n = 119) or to stop (n = 127) taking drugs temporarily for 7 days. The primary outcome was the modified Rankin Score at 90 days. Secondary outcomes included death, length of stay in hospital, discharge destination, activities of daily living, mood, cognition, and quality of life.

**Results:**

Blood pressure level (baseline 171/92 mmHg) fell in both groups but was significantly lower at 7 days in those patients assigned to continue antihypertensive drugs (difference 9.4/3.5 mmHg, *P* < .01). At 90 days, the primary outcome did not differ between the groups; the adjusted common odds ratio (OR) for worse outcome with continue versus stop drugs was .92 (95% confidence interval, .45-1.89; *P* = .83). There was no difference between the treatment groups for any secondary outcome measure, or rates of death or serious adverse events.

**Conclusions:**

Among patients with acute ICH, immediate continuation of antihypertensive drugs during the first week did not reduce death or major disability in comparison to stopping treatment temporarily.

## Introduction

High blood pressure (BP) is present in 75% of patients with acute intracerebral hemorrhage (ICH) and is substantially higher than premorbid levels.^1^, ^2^, ^3^ Raised BP occurs secondary to multiple factors, including neuroendocrine activation and increasing intracranial pressure, and is associated with a poor outcome.^4^, ^5^, ^6^, ^7^ More than 50% of patients with acute ICH are taking antihypertensive drugs before their stroke and hospital admission.

Although lowering BP long term after stroke is key for secondary prevention,^8^ it remains unclear whether prestroke antihypertensive drugs should be continued or stopped temporarily during the acute phase.^9^ Arguments both for and against each strategy can be postulated and guidelines lack firm recommendations related to this subject.^10^, ^11^ Continuing prior antihypertensive drugs after ICH might limit hematoma expansion, reduce the development of cerebral edema and early recurrence, and improve long-term outcome.^8^, ^12^ And yet, continuing treatment may lead to the development of hypotension, thereby compromising regional cerebral perfusion because of dysfunctional cerebral autoregulation.^13^ Further, continuing treatment involves administering tablets at a time when many patients have dysphagia and limited enteral access, a risk for aspiration pneumonia. Stopping treatment may result in secondary prevention being forgotten, thereby raising the risk of recurrent events and worsening outcomes long term.

Two trials have examined the question of whether prestroke BP drugs should be continued or stopped temporarily during the acute phase of stroke. The Continue or Stop Post-Stroke Antihypertensives Collaborative Study (COSSACS) found no difference in functional outcome, death, or serious adverse events, although it had low statistical power with only 763 participants recruited from a planned analysis of 2900 patients.^14^ No differential effect in patients with ICH versus ischemic stroke was reported. The Efficacy of Nitric Oxide in Stroke (ENOS) trial assessed the effects of glyceryl trinitrate (GTN) versus no GTN in 4011 participants with acute stroke; patients who were taking prestroke antihypertensive medications were also randomized to continue or stop these for 7 days in a partial factorial design.^15^ Although ENOS was neutral for both interventions,^16^ a subgroup of patients randomized to continue treatment within 12 hours had a worse functional outcome (unpublished data), an effect also seen in a meta-analysis of individual patient data from COSSACS and ENOS combined (Woodhouse et al, unpublished). Here, we report the results of a preplanned subgroup analysis of patients with ICH enrolled in ENOS and who were randomized to continue versus stop prestroke antihypertensive therapy,^17^ including those randomized within 12 hours of stroke onset.

## Methods

### ENOS Trial

Details of the ENOS study protocol, statistical analysis plan, patient characteristics at baseline, and main results have been published (ISRCTN99414122).^15^, ^16^, ^17^, ^18^ In brief, ENOS was a prospective, international, multicenter, randomized, blinded endpoint trial recruiting patients within 48 hours of ischemic stroke or ICH. Patients aged over 18 years with systolic blood pressure (SBP) level of 140-220 mmHg and who did not have a definite need for, or contraindication to, BP-lowering treatment were eligible. Randomization was performed centrally by computer to GTN (5 mg each morning) or no GTN, and, where relevant, to continue or stop taking prior antihypertensive drugs temporarily for 7 days. Randomization included stratification by stroke type (ICH versus ischemic stroke or stroke of unknown type) and minimization on key prognostic variables. GTN and no GTN were given with the patient masked to treatment; prestroke antihypertensive drugs were given open label.

In participants assigned to continue antihypertensive drugs, medication was administered orally, and those with dysphagia received treatment through a nasogastric feeding tube. If oral or tube feeding was not possible, treatment was withheld until feasible. Open-label antihypertensive drugs could be administered during the treatment period according to clinical need. During treatment, BP was measured once daily using a validated automatic clinical monitor (Omron HEM-705 CP or HEM-757; OMRON Healthcare Company, Kyoto, Japan)^19^ with a cuff of suitable size. After day 7, antihypertensive therapy that had been stopped was restarted according to clinical need. The trial protocol was approved by the national ethics committee in each participating country and all patients or surrogates gave informed consent. In this subgroup analysis, we included all patients with ICH recruited into the continue versus stop prestroke antihypertensive drugs part of ENOS.

### Outcomes

The primary outcome was the modified Rankin Scale (mRS scores: 0 = no residual disability, 5 = bedbound and requiring 24-hour care, 6 = death^20^) assessed at day 90. Key secondary outcomes were activities of daily living (Barthel Index scores: 0 = severe disability to 100 = no disability^21^), cognition (modified telephone Mini-Mental State Examination [t-MMSE] scores: 0 = severe dementia to 18 = normal^22^; Telephone Interview for Cognition Scale [TICS-M] scores: 0 = severe dementia to 37 = normal^23^; verbal fluency as animal naming scores: 0 = none to infinity), health-related quality of life (European Quality of Life-5 Dimensions [EQ-5D],^24^ from which health utility status [HUS] was calculated, scores: −.594 = very poor, 0 = death to 1.0 = perfect; European Quality of Life-Visual Analogue Scale [EQ-VAS] scores: 0 = very poor to 100 = excellent), and mood (short Zung depression Score [ZDS] scores: 0 = normal to 100 = severe depression^25^). The final follow-up was by telephone interview conducted by trained investigators blinded to treatment assignment at the trial-coordinating center. A postal questionnaire covering the outcome measures was sent if the patient could not be contacted. Safety outcomes included death, early neurological deterioration (defined as a decrease of at least 5 points on the Scandinavian Stroke Scale (SSS) from baseline to day 7 and/or a decrease in consciousness of more than 2 points on the SSS consciousness domain), recurrent stroke by day 7, hypotension (requiring intervention such as leg elevation or administration of fluids), hypertension (requiring treatment to lower BP), and serious adverse events. Serious adverse events whether related to treatment (definite, uncertain, no causality, unknown) and the systems affected by the adverse event were recorded by the local investigators.

### Imaging

For each patient, scan images were adjudicated centrally by expert neuroradiologists and trained physicians masked to clinical data and treatment allocation. Collected information included the presence of location of hemorrhage in the brain, an estimate of size and the presence of mass effect, atrophy, white matter disease, and old infarct or hemorrhage using validated scoring tools.^26^, ^27^ Hematoma parameters including volume, shape, density, shape index, density index, and presence of blood in the ventricles were also measured.^28^, ^29^, ^30^, ^31^, ^32^, ^33^, ^34^

### Analyses

As for the mRS and HUS, which have a separate category for death, we assigned an extreme score for death when analyzing each of the other outcome scale. The values used were −5: Barthel Index score; −1: EQ-VAS, SSS, t-MMSE, TICS-M, and verbal fluency; 0: EQ-5D/HUS; and 102.5: ZDS.^16^, ^17^ Comparisons were performed with binary logistic regression (dichotomous data), Cox regression (death), ordinal logistic regression (ordered categorical data),^35^ or multiple linear regression (continuous data). Analyses were adjusted for prognostic covariates: age, sex, premorbid mRS score, history of previous stroke, history of diabetes, severity (SSS), stroke syndrome (total anterior circulation versus other), SBP, feeding status, time to randomization, and treatment assignment (GTN versus no GTN). Heterogeneity of the treatment effect was assessed by including an interaction term in the adjusted statistical model for each of the following predefined subgroups: age, time to randomization, presence of ipsilateral carotid stenosis, number of prestroke antihypertensive drugs, feeding status, stroke severity, BP level at the time of randomization, feeding status, and treatment with GTN or no GTN. Analysis was performed using SPSS software version 22 (SPSS Statistics, Chicago, IL) on an Apple iMac computer (Apple Inc, Cupertino, California, USA). *P* values less than .05 were considered as statistically significant.

## Results

Recruitment into ENOS ran between July 2001 and October 2014. During this period, 629 patients with ICH were recruited into the trial, with 246 patients randomized to continue (n = 119) or stop (n = 127) antihypertensive drugs (Table 1). Thirty-nine patients were randomized within 12 hours (continue 18, stop 21). The treatment groups were well matched at baseline, with a mean age of 69 years, were male 59%, and had a mean BP level of 171/92 mmHg and a SSS severity score of 29.6 (National Institutes of Health Stroke Scale score of ~13.0 with mean ICH volume of 12.0 cm^3^). Most hematomas (89%) were located primarily in the deeper brain regions and many patients had leukoaraiosis (70%) and/or evidence of a previous stroke (51%) (Table 2); 72% of neuroimaging was performed within 12 hours of stroke onset.

### BP

**Figure 1:**
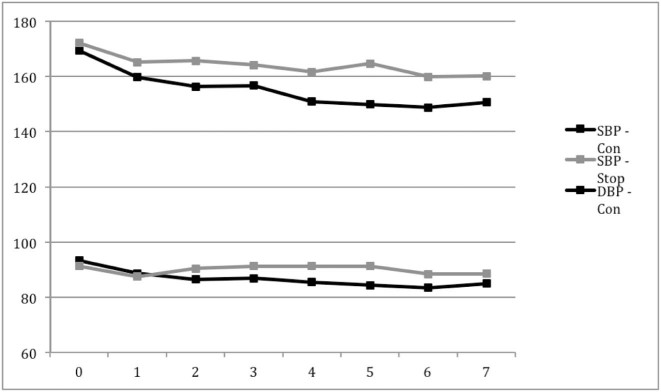
Blood pressure levels in patients with intracerebral hemorrhage who were randomized to continue or stop prestroke antihypertensive drugs. Day 0 is at randomization; day 1 is 2 hours post randomization. Comparisons by independent *t*-test at each time point (with Bonferroni correction) and repeated analysis of variance: *P* value less than .01/.01. Both SBP and DBP had significantly diverged by day 4 (2*P* = .010/2*P* < .026). Abbreviations: DBP, diastolic blood pressure; MD, mean difference in SBP and DBP for the continue versus stop groups; SBP, systolic blood pressure.

The mean BP level was 171/92 mmHg at baseline and declined in both groups over the 7 days of randomized treatment; by day 7, the BP level was lower by 9/4 mmHg in the group randomized to continue treatment (Fig 1). The baseline BP level was 176/97 mmHg in patients randomized at 12 hours and was lower by 28/16 mmHg at day 7 in those continuing treatment (Fig S1).

### Clinical Outcomes

**Figure 2:**
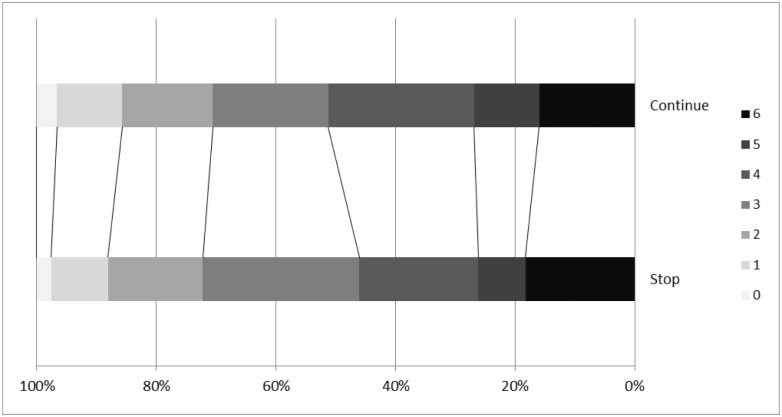
Distribution of modified Rankin scores at day 90 in patients randomized to continue versus stop prestroke antihypertensive drugs. Comparison by ordinal logistic regression with adjustment: common odds ratio .96 (95% CI, .60-1.51, *P* = .84). Abbreviation: CI, confidence interval.

**Figure 3:**
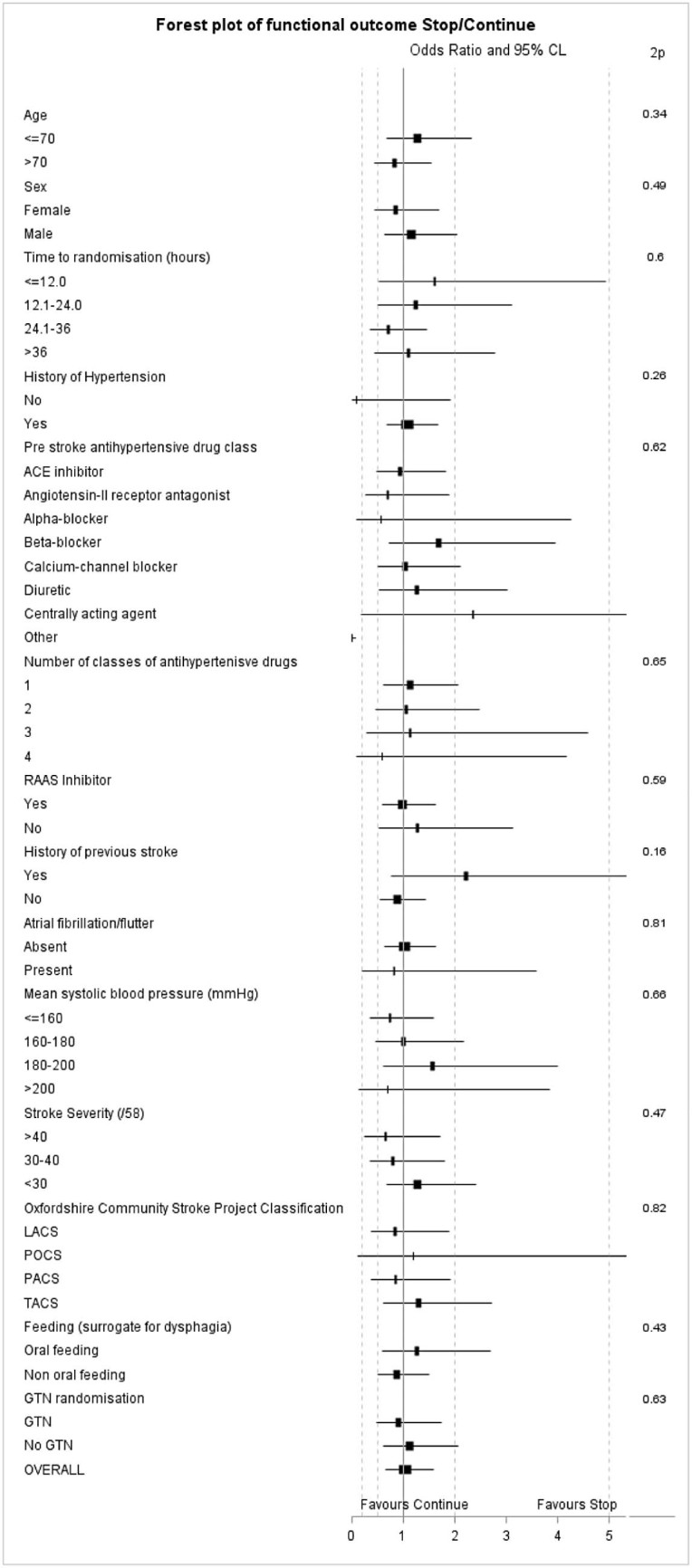
Subgroup analysis on the effects of functional outcome at day 90: continue versus stop. 2*P* is test for interaction. Abbreviations: ACE-I, angiotensin-converting enzyme inhibitor; GTN, glyceryl trinitrate; LACS, lacunar syndrome; PACS, partial anterior circulation syndrome; POCS, posterior circulation syndrome; RAAS, renin–angiotensin–aldosterone system; TACS, total anterior circulation syndrome.

There was no difference in mRS score between the treatment groups at day 90, common odds ratio for worse outcome in the continue group .92 (95% confidence interval, .45-1.89; *P* = .83) (Fig 2). A test of “goodness of fit” showed no evidence that the assumption of proportional odds had been violated (*P* = .07). There were no significant interactions between the effect of randomized treatment on mRS score in preselected subgroups (Fig 3). Additionally, there was no difference in mRS score in patients randomized within 12 hours to continue versus to stop taking prestroke BP drugs (Fig S2). The rates of clinical hypotension and hypertension were similar between the 2 groups (Table 3). There were no significant differences between the 2 groups at day 90 in any of the secondary clinical outcomes (Table 3) or death (Fig S3), or serious adverse rates (Table S1).

## Discussion

In this preplanned subgroup analysis of patients in ENOS with acute ICH, there was no difference in the primary outcome of function between patients randomized to 7 days of continuing versus stopping prestroke antihypertensive therapy^17^; this finding was consistent across all prespecified subgroups of patients. Similarly, there were no differences in safety outcomes or secondary outcome measures at day 90.

The overall neutral results seen for the continue versus stop comparison in ENOS (including both ischemic stroke and ICH)^16^ are similar to those seen in the smaller COSSACS trial.^14^ COSSACS recruited only 38 patients with ICH and has not reported these results separately; hence, this subgroup cannot be compared with the present substudy. Nevertheless, the 2 trials differed in several key aspects including time window for recruitment, exclusion or inclusion of dysphagia (and so differences in baseline severity), baseline BP level, length of treatment, and timing of measurement of the primary end point. In an individual patient data meta-analysis of COSSACS and ENOS combined, no difference in mRS score was seen in 284 patients with ICH who were randomized to continue versus to stop taking prestroke antihypertensive therapy (Woodhouse et al, unpublished). In that meta-analysis, mRS score was worse in patients randomized to continue treatment, irrespective of stroke type, if enrolled within 12 hours of onset. This finding was present in ENOS (unpublished data) but is not replicated in the present substudy, presumably due to the small number of patients and wide confidence intervals. BP lowering might reduce death or major disability if treatment is started within 6 hours of stroke.^37^, ^38^, ^39^ However, the present analysis assessed the issue of continuing or stopping pre-existing antihypertensive treatment (where differences in BP level between the treatment groups take days to develop), whereas the other trials initiated antihypertensive treatment in the hyperacute phase of stroke. Because it took several days for BP to differ between the randomized groups, it appears that short-term high BP level, as occurred in the group that were randomized to stop treatment temporarily, is not detrimental providing the difference occurs after the hyperacute period. Importantly, this observation appears to apply to those recruited within 12 hours where the BP level was higher by 28/16 mmHg at 1 week in those stopping treatment temporarily, a BP difference that was not associated with a worse outcome.

The strengths of the present study are 2-fold. First, it assessed a broad population of patients with ICH, with international enrollment from multiple race–ethnicity groups, and included patients with a wide range of severity, including those with dysphagia. Second, the data come from a high-fidelity trial with blinded assessment of outcomes, independent and masked adjudication of events, and near-complete follow-up.^16^ However, 2 limitations should be noted. First, ENOS excluded patients with very high BP and reduced consciousness (GCS < 8) or without motor signs. As a result, patients with large hemorrhages may have been under-represented. Second, fewer than 5% of patients had bleeding into the posterior fossa and therefore the results cannot be extrapolated to a population with posterior fossa hemorrhages. Recent observational data have shown that the BP rise is steeper, and final levels higher, in such patients as compared with lobar hemorrhages.^3^

In conclusion, this subgroup analysis of ENOS was neutral and did not identify any beneficial effects in continuing prestroke antihypertensive drugs in patients during the first week after acute ICH. Although BP lowering reduces chronic stroke recurrence, the present results suggest it is reasonable to withhold antihypertensive drugs taken before the onset of ICH until patients are neurologically stable and appropriate enteral or oral access has been established.

## Figures and Tables

**Table 1:** Baseline clinical characteristics of 246 patients with intracerebral hemorrhage and those randomized within 12 hours

Characteristics	All	Continue	Stop	12 h or less	Continue	Stop	2*P*
Number of patients (N)	246	119	127	39	18	21	
Country, United Kingdom (%)	138 (56.1)	68 (57.1)	70 (55.1)	20 (51.3)	10 (55.6)	10 (47.6)	.52
Age (years)	69.2 (11.5)	68.8 (11.3)	69.6 (11.6)	68.3 (13.0)	67.1 (13.7)	69.3 (12.6)	.58
Sex, male (%)	144 (58.5)	70 (58.8)	74 (58.3)	25 (64.1)	13 (72.2)	12 (57.1)	.44
Smoking, current (%)	37 (15.5)	20 (17.4)	17 (13.8)	4 (10.8)	2 (11.8)	2 (10.0)	.19
Premorbid mRS score > 0 (%)	81 (32.9)	43 (36.1)	38 (29.9)	9 (23.1)	5 (27.8)	4 (19.0)	.23
Previous stroke (%)	45 (18.3)	20 (16.8)	25 (19.7)	3 (7.7)	0	3 (14.3)	.06
Prior antihypertensive (%)	241 (98.0)	116 (97.5)	125 (98.4)	39 (100.0)	18 (100.0)	21 (100.0)	.33
Number of BP drugs (%)							
0	3 (1.2)	1 (.8)	2 (1.6)	—	—	—	—
1	107 (43.5)	55 (46.2)	52 (40.9)	20 (51.3)	13 (72.2)	7 (33.3)	.22
2	58 (23.6)	28 (23.5)	30 (23.6)	9 (23.1)	3 (16.7)	6 (28.6)	.47
3	25 (10.2)	8 (6.7)	17 (13.4)	5 (12.8)	1 (5.6)	4 (19.0)	1.00
4	11 (4.5)	4 (3.4)	7 (5.5)	2 (5.1)	1 (5.6)	1 (4.5)	1.00
5	1 (.4)	1 (.8)	0	—	—	—	—
Treated BP agent (%)							
ACE-I	106 (43.1)	51 (42.9)	55 (43.3)	17 (43.6)	6 (33.3)	11 (52.4)	.29
ARA	49 (19.9)	24 (20.2)	25 (19.7)	5 (12.8)	1 (5.6)	4 (19.0)	.35
Beta-receptor antagonist	70 (28.5)	26 (21.8)	44 (34.6)	10 (25.6)	3 (16.7)	7 (33.3)	.73
Calcium channel blocker	95 (38.6)	43 (36.1)	52 (40.9)	18 (46.2)	5 (27.8)	13 (61.9)	.12
Centrally acting agent	8 (3.3)	4 (3.4)	4 (3.1)	2 (5.1)	1 (5.6)	1 (4.8)	1.00
Diuretic	63 (25.6)	34 (28.6)	29 (22.8)	11 (28.2)	7 (38.9)	4 (19.0)	.53
Alpha-receptor antagonist	13 (5.3)	8 (6.7)	5 (3.9)	3 (7.7)	2 (11.1)	1 (4.8)	1.00
Other	3 (1.2)	1 (.8)	2 (1.6)	0	—	—	—
Previous high BP (%)	238 (96.7)	115 (96.6)	123 (96.9)	39 (100.0)	18 (100.0)	21 (100.0)	.21
Diabetes mellitus (%)	49 (19.9)	22 (18.5)	27 (21.3)	10 (25.6)	4 (22.2)	6 (28.6)	.33
Ischemic heart disease (%)	39 (15.9)	18 (15.1)	21 (16.5)	4 (10.3)	1 (5.6)	3 (14.3)	.58
Atrial fibrillation (%)	28 (11.4)	13 (10.9)	15 (11.8)	5 (12.8)	1 (5.6)	4 (19.0)	.75
TACS (%)	89 (36.2)	44 (37.0)	45 (35.4)	10 (25.6)	8 (44.4)	2 (9.5)	.15
SSS score (/58)	29.6 (12.6)	28.5 (12.3)	30.6 (12.9)	31.3 (11.4)	27.4 (12.4)	34.6 (9.4)	.37
NIHSS score (/42), calculated^36^	13.0 (5.4)	13.4 (5.3)	12.5 (5.5)	12.2 (4.9)	13.9 (5.4)	10.8 (5.4)	.37
Glasgow Coma Scale score (/15)	15 (1)	15 (1)	15 (1)	15 (1)	15 (1)	15 (0)	.13
SBP (mmHg)	170.8 (18.4)	169.5 (16.9)	172.1 (19.7)	175.8 (17.9)	175.0 (16.6)	176.4 (19.3)	.07
DBP (mmHg)	92.3 (13.4)	93.3 (14.0)	91.4 (12.8)	96.5 (11.7)	95.6 (14.1)	97.3 (9.5)	.031
Heart rate (bpm)	77.4 (15.8)	77.9 (15.9)	77.0 (15.9)	79.2 (17.9)	80.3 (15.1)	78.2 (20.3)	.22
Feeding status							
Normal diet	77 (31.3)	43 (36.1)	34 (26.8)	15 (38.5)	6 (33.3)	9 (42.9)	.28
Soft diet	58 (23.6)	22 (18.5)	36 (28.3)	10 (25.6)	7 (38.9)	3 (14.3)	.09
Nasogastric tube feeding	15 (6.1)	7 (5.9)	8 (6.3)	2 (5.1)	1 (5.6)	1 (4.8)	1.00
Percutaneous feeding tube	0	—	—	—	—	—	—
IV/SC fluids	45 (18.3)	20 (16.8)	25 (19.7)	4 (10.3)	2 (11.1)	2 (9.5)	1.00

Abbreviations: ACE-I, angiotensin-converting enzyme inhibitor; ARA, angiotensin receptor antagonist; BP, blood pressure; bpm, beats per minute; DBP, diastolic blood pressure; IV, intravenous; mRS, modified Rankin Scale; NIHSS, National Institutes of Health Stroke Scale; SBP, systolic blood pressure; SC, subcutaneous; SSS, Scandinavian Stroke Scale; TACS, total anterior circulation syndrome.

Data are number (%), mean (standard deviation), or median (interquartile range). Comparison of patients randomized within 12 hours versus those later by the Fischer exact test, the Mann–Whitney *U*-test, or *t*-test.

**Table 2:** Baseline neuroimaging characteristics of 246 patients with ICH and those randomized within 12 hours

Neuroimaging parameters	All	Continue	Stop	12 h or less	Continue	Stop	2*P*
Participants with a scan available	234 (95.1)	115 (96.6)	119 (93.7)	35 (89.7)	17 (94.4)	18 (85.7)	
Time, onset to neuroimaging (%)							
Less than 12 h	169 (72.2)	84 (73.0)	85 (71.4)	33 (94.3)	17 (100.0)	16 (88.9)	.89
12-24 h	38 (16.2)	19 (16.5)	19 (16.0)	1 (2.9)	—	1 (5.6)	—
More than 24 h	27 (11.5)	12 (10.4)	15 (12.6)	1 (2.9)	—	1 (5.6)	—
Location of hematoma (%)							
Lobar*	18 (7.7)	6 (5.2)	12 (10.1)	3 (8.6)	2 (11.8)	1 (5.6)	.41
Deep†	207 (88.5)	107 (93.0)	100 (84.0)	32 (91.4)	15 (88.2)	17 (94.4)	.10
Posterior‡	9 (3.8)	2 (1.7)	7 (5.9)	—	—	—	.28
ICH volume, ABC/2 (cm^3^)^34^	12.1 (14.0)	11.5 (13.6)	12.8 (14.5)	13.1 (17.0)	21.1 (20.5)	5.2 (6.3)	.67
IVH volume (cm^3^)	3.1 (4.6)	3.1 (4.5)	3.0 (4.8)	3.9 (3.4)	5.1 (3.5)	1.8 (2.3)	.61
Longest diameter (cm)	3.2 (1.4)	3.2 (1.3)	3.3 (1.5)	3.3 (1.5)	3.8 (1.6)	2.8 (1.2)	.76
Visual ICH size category (cm)^26^							
Less than 3	113 (49.1)	53 (46.9)	60 (51.3)	17 (48.6)	5 (29.4)	12 (66.7)	.25
3-5	80 (34.8)	42 (37.2)	38 (32.5)	10 (28.6)	6 (35.3)	4 (22.2)	
5-8	35 (1.2)	17 (15.0)	18 (15.4)	8 (22.9)	6 (35.3)	2 (11.1)	
More than 8	2 (.9)	1 (.9)	1 (.9)	—	—	—	
Shape (/5)^29^	3.3 (1.3)	3.4 (1.5)	3.2 (1.4)	3.4 (1.5)	4.0 (1.4)	2.8 (1.3)	.69
Index^31^	1.7 (3.1)	1.8 (4.0)	1.6 (1.7)	1.7 (1.3)	2.2 (1.4)	1.3 (1.1)	.34
Density (/5)^29^	2.7 (1.4)	2.6 (1.3)	2.8 (1.4)	2.7 (1.4)	2.8 (1.4)	2.5 (1.4)	.84
Index^30^	.2 (.1)	.2 (.1)	.2 (.1)	.2 (.1)	.2 (.1)	.2 (.1)	.34
Graeb score^32^	3.4 (2.2)	3.3 (2.1)	3.4 (2.3)	4.9 (2.1)	6.2 (.8)	2.7 (1.5)	.033
Modified^33^	5.0 (4.4)	4.9 (3.5)	5.2 (4.8)	8.0 (4.4)	10.4 (3.5)	4.0 (2.0)	.034
Leukoaraiosis (%)	171 (73.1)	82 (71.3)	89 (78.8)	21 (60.0)	10 (58.8)	11 (61.1)	1.00
Mass effect (%)							
No swelling to mild swelling (%)	98 (41.9)	47 (40.9)	51 (42.9)	17 (48.6)	5 (29.4)	12 (66.7)	.55
Moderate to severe swelling (%)	33 (14.1)	18 (15.7)	15 (12.6)	18 (51.4)	12 (70.6)	6 (33.3)	.14
Previous stroke lesion (%)	126 (53.8)	60 (52.2)	66 (55.5)	20 (57.1)	10 (58.8)	10 (55.6)	.06
Brain tissue reduction (%)	153 (65.4)	77 (67.0)	76 (63.9)	22 (62.9)	12 (70.6)	10 (55.6)	.42
Cortical atrophy (%)	119 (50.9)	58 (50.4)	61 (51.3)	15 (42.9)	6 (35.3)	9 (50.0)	.70
Central atrophy (%)	146 (62.4)	72 (62.6)	74 (62.2)	22 (62.9)	12 (70.6)	10 (61.1)	.65

Abbreviations: ACA, anterior cerebral artery; ICH, intracerebral hemorrhage; IVH, intraventricular hemorrhage; MCA, middle cerebral artery; PCA, posterior cerebral artery.

Data are number (%) or mean (standard deviation). Comparison of patients randomized within 12 hours with those randomized later by the Fisher exact test, the Mann–Whitney *U*-test, or *t*-test.

Shape index = hematoma perimeter/4∏ × surface area.

Density index = standard deviation/mean of Hounsfield units.

* Lobar: ICH centered on border-zone regions, ACA, PCA territory, and MCA territory, excluding striatocapsular regions.

† Deep: ICH centered on lacunar, MCA territory including striatocapsular regions.

‡ Posterior: ICH centered on the cerebellum and/or the brain stem.

**Table 3:** Primary and secondary outcomes at days 7 and 90: continue versus stop prestroke antihypertensive drugs

Outcome	N	All	Continue	Stop	Unadjusted OR/MD (95% CI)	2*P*	Adjusted OR/MD (95% CI)	2*P*
Day 7 (or discharge)		246	119	127				
Death (%)	246	6 (2.4)	2 (1.7)	4 (3.2)	.53 (.09-2.92)	.46	.47 (.07-3.01)	.58
SSS (/58)	244	33.2 (16.1)	33.1 (16.3)	33.3 (15.9)	−.3 (−4.3-3.8)	.90	−.8 (−4.0-2.5)	.64
Recurrent stroke (%)	245	6 (2.5)	3 (2.5)	3 (2.4)	1.06 (.21-5.36)	.37	1.01 (.18-5.92)	.99
SBP (mmHg)	210	155.4 (26.0)	150.6 (26.4)	160 (24.9)	**−6.2 (−12.2 to −.2)**	**.043**	**−7.5 (−14.7 to −.3)**	**.037**
Hypotension (%)	246	3 (1.2)	2 (1.7)	1 (.8)	2.15 (.19-24.07)	.53	.09 (.00-6.06)	.26
Hypertension (%)	246	36 (14.6)	15 (12.6)	21 (16.5)	.73 (.36-1.49)	.39	.77 (.57-2.92)	.54
Hospital events		244	118	126				
Died in hospital (%)	244	28 (11.5)	14 (11.9)	14 (11.1)	1.08 (.49-2.37)	.86	1.03 (.35-2.38)	.85
Hospital stay (days)	244	11 [7,33]	12 [7,33]	11 [7,27]	−1.67 (−8.38-5.03)	.62	−.68 (−7.09-5.72)	.83
Death or institution (%)	244	105 (43.0)	51 (43.2)	54 (42.9)	.76 (.45-1.27)	.29	.69 (.38-1.24)	.22
Day 90			119	126				
Death (%)	245	42 (17.1)	19 (16.0)	23 (18.3)	.85 (.44-1.66)	.64	.82 (.37-1.82)	.72
mRS score (/6)	245	3.5 (1.7)	3.5 (1.7)	3.5 (1.6)	1.0 (.7-1.6)	.94	1.0 (.7-1.6)	.86
BI	245	57.4 (39.8)	57.1 (39.8)	57.6 (40.0)	−.6 (−10.6-9.5)	.91	−3.2 (−11.7-5.3)	.45
t-MMSE	141	9.1 (7.43)	9.0 (7.4)	9.2 (7.5)	−.2 (−2.7-2.3)	.89	−1.1 (−3.2-.9)	.28
TICS-M	130	18.9 (15.9)	19.2 (15.9)	18.6 (15.9)	.7 (−4.9-6.2)	.82	−1.4 (−6.1-3.3)	.55
Animal naming (/infinity)	136	7.2 (7.5)	7.2 (7.3)	7.2 (7.7)	−.6 (−3.1-1.9)	.64	−.6 (−3.3-2.1)	.66
ZDS (/100)	197	64.3 (24.0)	64.1 (23.4)	64.4 (24.7)	−.3 (−7.1-6.4)	.92	1.7 (−4.2-7.7)	.57
EQ-5D/HUS (/1)	244	.42 (.31)	.40 (.30)	.43 (.33)	−.03 (−.11-.05)	.52	−.04 (−.11-.03)	.24
EQ-VAS (/100)	213	50.1 (31.5)	50.9 (31.0)	49.4 (32.1)	1.6 (−7.0-10.1)	.72	−1.9 (−9.6-5.9)	.64

Abbreviations: BI, Barthel Index; EQ-5D, European Quality of Life-5 Dimensions; EQ-VAS, European Quality of Life-Visual Analogue Scale; HUS, health utility status; ICH, intracranial hemorrhage; MD, mean difference; mRS, modified Rankin Scale; OR, odds ratio; t-MMSE, Modified telephone Mini-Mental State Examination; SBP, systolic blood pressure; SSS, Scandinavian Stroke Scale; TICS-M, Modified Telephone Interview for Cognitive Status; t-MMSE, telephone Mini-Mental State Examination; VAS, Visual Analogue Scale; ZDS, Zung Depression Scale.

Data are the number of patients (%), median (interquartile range), or mean (standard deviation). Comparison by logistic regression, ordinal regression, or multiple regression, shown as OR or MD, with adjustment for age, sex, premorbid mRS score, history of previous stroke, history of diabetes, stroke severity, stroke syndrome (total anterior circulation), SBP, feeding status, time to randomization, and treatment assignment (glyceryl trinitrate versus none).

Range of scores: SSS: −1 (death) to 0 (coma with quadriplegia) to 58 (normal neurological status); BI: −5 (death) to 0 (severe disability) to 100 (no disability); modified t-MMSE: −1 (death), 0 (severe dementia) to 18 (normal); TICS-M: −1 (death), 0 (severe dementia) to 37 (normal); verbal fluency (number of animals named in 1 minute): −1 (death), 0 (none named) to infinity; HUS (derived from EQ-5D): −.5 (very poor quality of life), 0 (death) to 1.0 (perfect quality of life); EQ-VAS: −1 (death), 0 (very poor) to 100 (excellent). ZDS: 0 (normal), 100 (severe depression) to 102.5 (death).

The numbers highighted in bold indicate that these values were statistically significant.
