# Supplementary material for: Continuing versus Stopping Prestroke Antihypertensive Therapy in Acute Intracerebral Hemorrhage: A Subgroup Analysis of the Efficacy of Nitric Oxide in Stroke Trial
Source: J Stroke Cerebrovasc Dis. 2016 May;25(5):1017–26. doi: 10.1016/j.jstrokecerebrovasdis.2016.01.010 (PMC4851456; doi:10.1016/j.jstrokecerebrovasdis.2016.01.010)
Supplement: Appendix S1 — Table S1 and Figs S1-S3. [file mmc1.docx]

**Continue versus stopping pre-stroke antihypertensive therapy in acute intracerebral haemorrhage: a subgroup analysis of the Efficacy of Nitric Oxide in Stroke (ENOS) trial**

Kailash Krishnan MRCP,^1^ Polly Scutt MSc,^1^ Lisa Woodhouse MSc,^1^ Alessandro Adami MD,^2^ Jennifer L Becker MD FRCR,^3^ Lesley A Cala MD FRCR,^4^ Ana M Casado MD,^5^ Christopher Chen MRCP FAMS,^6^ Robert A Dineen PhD FRCR,^7^ John Gommans FRACP,^8^

Panos Koumellis MRCP FRCR,^9^ Hanna Christensen MD PhD,^10^ Ronan Collins MD FRCPI,^11^ Anna Czlonkowska MD PhD,^12^ Ken Lees MD, FRCP,^13^ George Ntaios MD PhD,^14^ Serefnur Ozturk MD,^15^ Stephen J Phillips MBBS FRCPC,^16^ Nikola Sprigg DM MRCP,^1^ Szabolcs Szatmari MD,^17^Joanna M Wardlaw FRCR FMedSci,^5^ Philip M Bath DSc FRCP;^1^ for the ENOS Investigators.

Web Supplement containing additional table and figures.

**Web Table 1.** Serious adverse events at day 90 for continue versus stop pre-stroke antihypertensive drugs in 246 patients with intracerebral haemorrhage. Data are number of patients (%) and mean (standard deviation).

| Adverse event | Continue | Stop | 2p | Continue | Stop | 2p |
| --- | --- | --- | --- | --- | --- | --- |
|  |  | All |  |  | Fatal |  |
| Neurological † | 10 (8.4) | 14 (11.0) | 0.50 | 6 (5.0) | 8 (6.3) | 0.67 |
| Cardiac ‡ | 4 (3.4) | 10 (7.9) | 0.17 | 2 (1.7) | 2 (1.6) | 1.00 |
| Pulmonary embolism | 1 (0.8) | 2 (1.6) | 1.00 | 1 (0.8) | 0 (0.0) | 0.48 |
| Pneumonia | 8 (6.7) | 8 (6.3) | 0.89 | 6 (5.0) | 4 (3.1) | 0.53 |
| Other causes | 0 (0.0) | 1 (0.8) | 1.00 | - | - | - |
| Total | 27 (22.7) | 35 (27.6) | 0.38 | 6 (33.3) | 4 (19.0) | 0.46 |

† Includes complication of initial stroke, extension of initial stroke, symptomatic intracranial haemorrhage and recurrent stroke

‡ Includes myocardial infarction, sudden cardiac death and other cardiovascular causes

**Web Figure 1.** Blood pressure levels in patients with intracerebral haemorrhage who were randomised within 12 hours to continue or stop pre-stroke antihypertensive drugs. Day 0 is at randomisation; day 1 is 2 hours post-randomisation. MD is the mean difference in systolic and diastolic blood pressure for the continue versus stop groups. Comparisons by independent t test at each time point (with Bonferroni correction), and repeated analysis of variance: p<0.01/<0.01. Both systolic and diastolic blood pressure had significantly diverged by day 5 (p<0.001).

**Web Figure 2.** Distribution of modified Rankin scores at day 90 in 39 patients randomised within 12 hours to continue versus stop pre-stroke antihypertensive drugs. Comparison with Mann-Whitney U-test (p=0.43).

**Web Figure 3.** Comparison of survival between continue versus stop groups at day 90. Comparison using Cox proportional hazards model: hazard Ratio 1.01 (95% CI 0.81-1.27; p=0.88).
